# Ranibizumab therapy for predominantly hemorrhagic neovascular age-related macular degeneration

**DOI:** 10.14744/nci.2021.52323

**Published:** 2022-03-23

**Authors:** Ozlem Dikmetas, Sibel Kadayifcilar, Bora Eldem, Ulkar Feyzullayeva

**Affiliations:** Department of Ophthalmology, Hacettepe University Faculty of Medicine, Ankara, Turkey

**Keywords:** Age-related macular degeneration, hemorrhagic, ranibizumab

## Abstract

**Objective:**

Predominantly hemorrhage represents one of the possible manifestations of choroidal neovascularisation (CNV) in eyes with age-related macular degeneration (AMD). The purpose of this study is to evaluate the effecte of ranibizumab treatment in patients with predominantly hemorrhagic CNV secondary to AMD.

**Methods:**

Twenty-five patients with predominantly hemorrhagic choroidal neovascularization due to AMD with at least three ranibizumab injections and followed up for at least 12 months were included in the study. The months of follow-up were recorded (baseline, 3^rd^, 6^th^, and 12^th^ months). The change in central macular thickness (CMT) on optical coherence tomography, visual acuity (VA) in ETDRS letters, and lesion size on fundus fluorescein angiography were evaluated.

**Results:**

The mean age of the patients was 68.1±5.7 (range: 63–82) years, the mean follow-up was 19.9±14.5 (range: 12–67) months, and the mean number of injections was 4.0±1.4 (range: 3–15). The initial VA was 39.3±17.9 (range: 1–65) letters, CMT was 272.7±104 (range: 164–587) μm, and the initial lesion width was 11.4±10.5 (range: 1.3–45.7) mm^2^. The VA was 41.4±20.1 (range: 5–75) and 36.9±21.8 (range: 4–80) letters (p=0.150), CMT was 270.7±110 (range: 159–570) and 230.4±108 (range: 109–667) μm (p=0.009) and the lesion width was 10.9±11.5 (range: 1.1–39.7) and 10.4±11.6 (range: 1.2–44.3) mm^2^ at 6^th^ and 12^th^ month, respectively. No factor was found to be associated with final CMT.

**Conclusion:**

Although the final visual outcome is limited by the progression of the disease, hemorrhagic lesions treated with ranibizumab have stable anatomical outcome.

**A**ge-related macular degeneration (AMD) is a leading cause of blindness among adults in developed countries [1]. It is a degenerative disease of the macula that causes central vision loss. There are two types of AMD, namely dry (atrophic) and wet (neovascular) [2]. Neovascular AMD (nAMD) typically progresses rapidly [2]. Subretinal hemorrhage (SRH) represents one of the possible manifestations of choroidal neovascularisation (CNV) in eyes with nAMD [3]. The management of eyes with predominantly hemorrhagic CNV lesions (hemorrhage>50% of the lesion) is both complicated and controversial [3–5]. Treatments have been using for macular hemorrhage due to AMD, including tissue plasminogen acivator (TPA), anti-vascular endothelial growth factor (VEGF) drugs, intravitreal gas tamponades, photodynamic therapy, and vitrectomy [4–9]. However, no consensus has been reached as to which of these treatments is the most efficacious [10]. Intravitreal anti-VEGF treatment is currently considered the gold standard for the treatment of nAMD [11]. Interestingly, when the results of the MARINA and ANCHOR trials for predominantly hemorrhagic lesions were analyzed, the efficacy of ranibizumab, an anti-VEGF drug, was found to be unclear [12]. Moreover, the submacular surgery trials found no differences in terms of vision improvement without the treatment of the underlying haemorrhage [13]. The present study sought to determine the effect of ranibizumab on predominantly hemorrhagic neovascular AMD.

## Materials and Methods

Following Hacettepe University Faculty of Medicine Ethics Board approval (GO 18/90-21, 31.01.2018), data of patients who were treated in University School of Medicine Department of Ophthalmology’s Retina Unit. treatment for hemorrhagic nAMD between 2006 and 2017 were collected, retrospectively. The study was conducted according to the Declaration of Helsinki Principles. Patients with hemorrhagic CNV lesions secondary to nAMD and with at least 1 year of follow-up were included in the study. The other inclusion criteria were being at least 50 years of age and ≥50% of the lesion being hemorrhagic (as defined in the Macular Photocoagulation Study) [14, 15]. Hemorrhagic AMD was defined as ≥50% of the lesion being hemorrhagic with optical coherence tomography (OCT) or clinical fundus examination. The exclusion criteria for the study were the presence of CNV due to other eye diseases (e.g. inflammation) and a follow-up period of <1 year. All the patients received intravitreal injections of ranibizumab (0.5 mg/0.05 ml Lucentis; Genentech Inc., San Francisco, CA, USA). The intravitreal injections were given at baseline, at month 1 and at month 2. All eyes were injected in an operating room. Topical anesthesia was obtained, the conjunctiva was irrigated with 2.5% povidone iodine solution followed by scrub of the eyelids and periorbital area. Patients received an intravitreal injection of 0.5 mg ranibizumab via the pars plana, 3.5–4.0 mm posterior to the limbus using a syringe with a 30-gauge needle. After procedure, all patients instilled moxifloxacin for 5 days. After the 3 monthly injections, retreatment was done as needed (PRN) [16], as following: (1) Visual acuity (VA) loss of five letters or more with Early Treatment of Diabetic Retinopathy Study (ETDRS) letters in comparison with the VA of previous visit; (2) fluid or hemorrhage with clinical examination and increase in central thickness in OCT, recurrent fluid or novel fluid accumulation in OCT; (3) newly developed hemorrhage secondary to CNV; or (4) an active nAMD finding on the fundus fluorescein angiography (FFA), indocyanine green angiography (ICGA) or OCT images. The VA (ETDRS letters), the lesion size on the FFA images and the central macular thickness (CMT) on the OCT images were recorded for each patient at baseline as well as the 3^rd^, 6^th^, and 12^th^ months and then analyzed. The diameter of the lesion was analyzed using VISUPAC imaging software (Carl Zeiss Meditec AG, Jena, Germany). The CMT was measured on a horizontal and a vertical scan, and it was defined as the distance between the internal limiting membrane and the inner surface of the retinal pigment epithelium (RPE) at the foveal center. These two measurements were analyzed as the CMT. ICGA was performed for the differential diagnosis of polypoidal choroidal vasculopathy which is the most common cause of hemorrhagic CNV. All measurements were done by the same clinicians (OD).

**Highlight key points**

• Intravitreal injection of anti-VEGF drugs has become the standard treatment for patients with CNV secondary to AMD.
• Most multicenter controlled and randomized studies have excluded patients with hemorrhage occupying >50% of the lesion.
• Intravitreal ranibizumab treatment may improve the vision and anatomy of hemorrhagic AMD eyes without inducing major adverse effects.
• Intravitreal ranibizumab treatment may be an appropriate non-surgical alternative in patients with hemorrhagic AMD or, at least, may be utilized on a temporary basis to delay the need for invasive procedures.

All data were entered into a database and analyzed using the statistical package for social sciences, SPSS version 18 (SPSS Inc., Chicago, IL, USA). Mann Whitney U test and Chi-square test were employed. The association between lesion components (CMT, lesion width) and VA was analyzed Spearman correlation test. Shapiro-Wilk test for normality and graphics (qq plot, boxplot) were evaluated together to decide the distribution of the data (whether they show normal distribution). Frequency and percentage were used in categorical data, mean and standard deviation were used in numerical data. P<0.05 were considered to be statistically significant.

## Results

Twenty-five eyes of 25 patients were followed for at least 12 months from September 2006 to December 2017 and the resultant data were retrospectively evaluated. The mean age of the patients was 68.1±5.7 years (range: 63–82 years) and 48% of them were male (12 male and 13 female). The mean number of ranibizumab injections was 4.0±1.4 (range: 3–15) during the entire follow-up period and 3.8±1.6 (range: 3–9) during the first 12 months. The patients’ ocular characteristics are described in Table 1. The mean follow-up period was 19.9±14.5 months (range: 12–67 months). The mean VA at baseline (ETDRS letters) was 39.3±17.9 letters (range: 1–65 letters). Moreover, the mean CMT at baseline was 272.7±104 μm (range: 164–587 μm), while the mean lesion width at baseline was 11.4±10.5 mm^2^ (range: 1.3–45.7 mm^2^).

**Table 1.** Ocular characteristics of patients

	Baseline	3^th^ month	6^th^ month	12^th^ month
VA (ETDRS)±SD, letters (range)	39.3±17.9 (1–65)	42.1±22.8 (6–75)	41.4±20.1 (5–75)	36.9±21.8 (4–80)
Width of lesion±SD (range) mm^2^	11.4±10.5 (1.3–45.7)	11.7±11.5 (2.6–30)	10.9±11.5 (1.1–39.7)	10.4±11.6 (1.2–44.3)
CMT with OCT±SD (range) μm	272.7±104 (164–587)	254.1±120 (169–484)	270.7±110 (159–570)	230.4±108 (109–667)

SD: Standard deviation; VA: Visual acuity; ETDRS: Early treatment of diabetic retinopathy study; CMT: Central macular thickness; OCT: Optical coherence tomography.

At month 3, the mean VA (ETDRS letters) was 42.1±12.3 letters (range: 13–65 letters), the mean CMT was 254.1±120 μm (range: 169–484 μm) and the mean lesion width was 11.7±11.5 mm^2^ (range: 2.6–30 mm^2^). At month 6, the mean CMT was 270.7±110 μm (range: 159–570 μm), which indicated a non-statistically significant decrease when compared with baseline (p=0.165). Moreover, the mean lesion width at month 6 was 10.9±11.5 mm^2^ (range: 1.1–39.7 mm^2^), while the mean VA was 41.4±20.1 letters (range: 5–75 letters). The mean lesion width and VA measurements at month 6 were not found to be statistically different from the measurements at baseline (p=0.180 and p=0.134, respectively).

**Figure 1.**
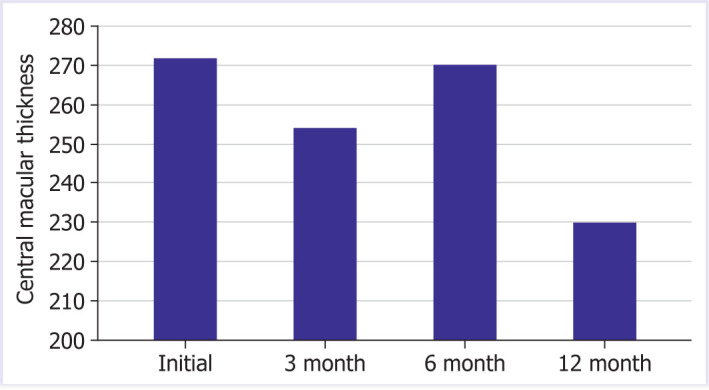
Mean change in central macular thickness over the 3-month, 6-month and 12-month follow-up.

**Figure 2.**
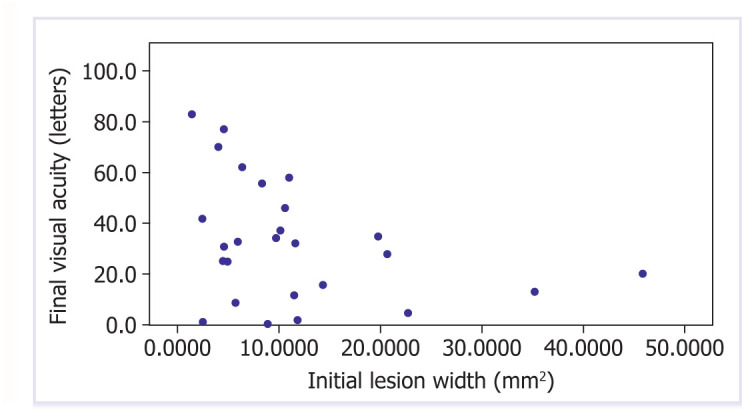
Dot plot graphics in the correlation between the initial lesion width and final visual acuity at 12^th^ month.

**Figure 3.**
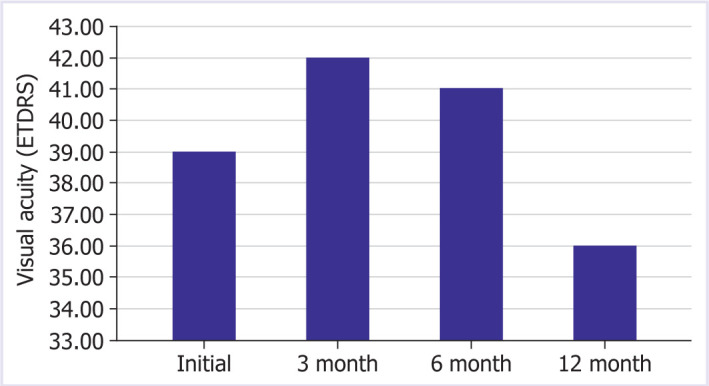
Change graphics in visual acuity over the 3-month, 6-month and 12-month follow-up.

**Figure 4.**
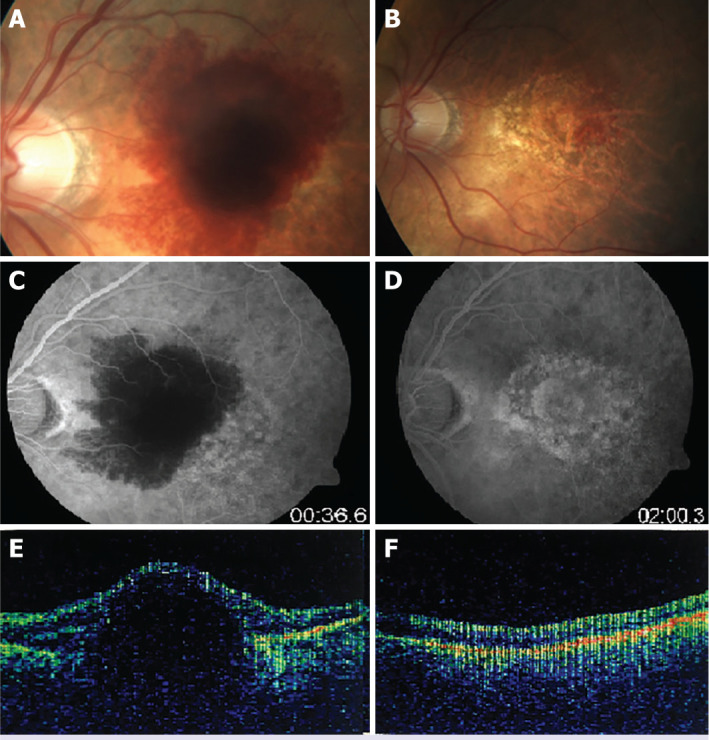
The patients with predominantly hemorrhagic choroidal neovascularization due to age-related macular degeneration Fundus photography of the patient pretreatment **(A)** And after treatment at 12^th^ month **(B)**. Fundus fluoressein angiography of the patient pretreatment **(C)** and after treatment at 12^th^ month **(D)**. Optical coherence tomography of the patient pretreatment **(E)** and after treatment at 12^th^ month **(F)**.

At the 12^th^ month, the mean CMT was 230.4±108 μm (range: 109–667 μm), which indicated a statistically significant decrease when compared with baseline (p=0.009). The mean change in the CMT as shown on the OCT images during the first 12 months is presented in Figure 1. The initial mean lesion width of 10.4±11.6 mm^2^ (range: 1.2–44.3 mm^2^) was found to be inversely correlated with the final VA (p=0.048, correlation coefficient=–0.371) (Fig. 2). The final mean VA was 36.9±21.8 letters (range: 4–80 letters), which was not statistically significantly different from the initial VA (p=0.150) (Fig. 3, 4).

Although 15 (60%) patients remained stable throughout the first 12 months, 10 (40%) patients did not respond to the treatment and developed wide scars. The VA was found to have improved by at least 15 letters in three (12%) patients, whereas it was found to have decreased by ≥15 letters in five (25%) patients at the 12-month follow-up point.

One patient developed a mild vitreous hemorrhage at the 5^th^ month (i.e. 2 months after the third injection), although the hemorrhage resolved spontaneously within a month, leaving behind a scar. There were not any complications. No systemic adverse events related to the usage of ranibizumab were recorded during the follow-up period.

## Discussion

The intravitreal injection of anti-VEGF drugs has become the standard treatment modality for patients with CNV secondary to AMD [11]. As the number of individuals with AMD increases, it is reasonable to expect that the incidence of submacular hemorrhage associated with AMD will also increase. Yet, most prior multicenter controlled and randomized studies have excluded patients with hemorrhages occupying >50% of the lesion [12].

A range of treatments can be used for the management of hemorrhagic AMD, all of which are associated with different outcomes [17]. For instance, when injected into the vitreous cavity, TPA can cause blood toxicity [18]. Hesse et al. [19] found an increase in SRH due to intravitreal TPA injection, while Kimura et al. [20] reported the complete liquefaction of acute SRH prior to surgery for haemorrhage. Heriot treated the SRH with gas and showed in the Vitrectomy meeting [21]. Ohji et al. found better VA to be achieved following the use of intravitreal gas [22, 23]. When SRH persists for longer than 2 weeks, surgical treatment modalities may be applied [13]. SRH could harm the photoreceptors and outer retinal layers [13, 24]. The surgical removal of hemorrhages related to AMD in the eyes is associated with a poor prognosis in terms of the recovery of VA [13]. Current surgical techniques allow for the removal of subretinal blood. The surgical results seen in AMD patients have often revealed a variable improvement in relation to VA, although the final VA has generally been poor in most series. The use of surgical techniques has not been found to increase the chances of stable or improved VA, and such techniques have been found to be associated with a high risk of rhegmatogenous retinal detachment [25]. Machemer et al. [26] analyzed the outcomes of the macular translocation procedure and found only one patient to exhibit increased final VA. Peyman et al. [27] evaluated the impact of an autologous RPE-choroid graft to the subretinal space and found VA to be increased as a result of the treatment. McLaren et al.[28] investigated an alternative way of performing macular translocation although they did not achieve very effective results [29]. Macular translocation had no promising results [29]. In light of these differing and inconclusive results, additional information is required concerning the best clinical approach for the treatment of SRH.

Anti-VEGF therapy represents an important therapeutic choice for the treatment of hemorrhagic AMD [30]. Stifter et al. [31] evaluated the effects of intravitreal bevacizumab treatment for haemorrhagic AMD and found that VA was improved in 100% of patients and improved by at least three lines in 9.5% of patients. The hemorrhage was found to be reduced at the 4-month follow-up point. The application of ranibizumab for the treatment of nAMD has been investigated in numerous clinical studies [3–5, 11, 32–34], although studies concerning the use of ranibizumab in relation to hemorrhagic AMD remain scarce. Moreover, there have been no randomized controlled trials of anti-VEGF therapy with regard to hemorrhagic AMD. Chang et al. [35] assessed the effect of ranibizumab on seven patients with hemorrhagic AMD and found subfoveal ﬁbrosis to occur. In the present study, we found the patients’ VA to be stable following anti-VEGF monotherapy in 25 eyes with predominantly hemorrhagic nAMD after 12 months of treatment. The baseline VA was not found to be associated with the CMT; however, a small lesion width at baseline was found to be associated with good VA at the 12-month follow-up point. In our study, the lesion width was found to be inversely associated with the final VA, although similar to Chang et al.’s findings, while the CMT decreased, the VA did not increase, possibly due to the occurrence of subretinal fibrosis. In fact, we observed subretinal fibrosis in 10 (40%) patients whose VA did not increase following the treatment. Iacono et al. [36] found that, at the 12-month follow-up point, the mean VA improved signiﬁcantly and the mean CMT decreased with the progressive resolution of macular bleeding in 22 out of 23 patients who were treated with ranibizumab. Iacono et al. investigated the effect of the baseline VA on patients’ response to ranibizumab treatment and determined that the letter gain was generally inversely correlated with the baseline VA. This finding is similar to the results of the MARINA and ANCHOR studies. Araiz et al. [37] concluded that the intravitreal administration of ranibizumab for the treatment of exudative hemorrhagic AMD significantly improved patients’ VA, decreased the lesion characteristics (drusen, macular hemorrhages, lipid exudates and retinal pigment epithelial detachment [PED]), and reduced the CMT after 12 months. A number of factors, including intraretinal cysts, the epiretinal membrane, and the architecture of the retinal layers, have been found to affect patients’ response to treatment, although the size of the hemorrhage has been found to be the most important factor in relation to poor results [12]. For instance, decreased long-term VA has been found to be associated with the thickness of the macular haemorrhage [12]. Avery et al. [38] described 41 eyes of 40 patients with hemorrhagic AMD who were followed up without surgical intervention. The authors found significant correlation between the initial size of the hemorrhage and the visual outcome at the 12- and 36-month follow-up points. In the present study, we observed similar results in terms of the initial lesion width. More specifically, the initial lesion width was found to be inversely correlated with the final VA (p=0.038, r=–0.371). Applying the treat and extend regime could be provide more achievements to these results. Treat and extend regime is widely accepted today [39]. Tanaka et al. [40] observed the development of new hemorrhagic vision-threatening lesions during anti-VEGF treatment in three eyes for 3.5 years and in one eye for more than 3.5 years. They concluded that the treatment of hemorrhagic AMD was difficult and that new studies could prove helpful.

It is important to acknowledge that the present study had several limitations. The major limitation of the study is its retrospective design. Moreover, due to the retrospective design, there was no control group included in the study. In addition, the study’s sample size was relatively small.

### Conclusion

In conclusion, this study showed that intravitreal ranibizumab treatment could improve the vision and anatomy of hemorrhagic AMD eyes without inducing major adverse effects. As a result, such treatment could be an appropriate non-surgical choice for patients with haemorrhagic AMD or, at least, serve to delay the need for surgical intervention. Further prospective studies involving larger population sizes are suggested to analyze the long-term effects of intravitreal ranibizumab on eyes with hemorrhagic AMD.
